# Demand response with heat pumps: Practical implementation of three different control options

**DOI:** 10.1177/01436244221145871

**Published:** 2022-12-13

**Authors:** Jenny Crawley, Adria Martin-Vilaseca, Jez Wingfield, Zachary Gill, Michelle Shipworth, Clifford Elwell

**Affiliations:** 1 UCL Energy Institute, 4919 University College London, London, UK; 2 SOAP Retrofit, Bristol, UK

**Keywords:** Case study, demand response, empirical, flexibility, heat pumps

## Abstract

The electrification of heating and transport and decarbonisation of supply creates a need for demand side flexibility to balance the grid. Heat pumps are expected to form a major part of heat delivery, and many modelling studies have investigated the technical potential of heat pump demand response. However, little empirical work has been reported on the practical implementation of such demand response in occupied homes. This paper presents a cross-case comparison of three early adopters of heat pump demand response in the UK. The aim was to reduce heat pump electricity consumption during the same peak period, but each employed a different control strategy: lowered air temperature setpoints, lowered flow temperature and blocked heat pump compressor. A 56–90% electricity reduction during the peak period was observed; the success of the demand response depended on how the control strategy affected the heat pump and the rest of the heating system. However, no one stakeholder is responsible for all these system components. The fabric, heating distribution and control system and heat pumps installed are highly heterogeneous across the stock, highlighting that flexibility mechanisms must be developed that can be tailored to or work across their range.

**Practical application:**

Three case studies of different heat pump demand response control strategies in real homes are presented. All three households reduced their electricity consumption during a peak period but delivered unintended consequences where the heat pump’s logic did not correspond to the demand response requirements. This study highlights that the implementation of heat pump demand response to support electricity system operation requires a clear definition of electricity system need as well as practical demand response mechanisms to be integrated into heating system design.

## Introduction

A growing number of countries have pledged to achieve net zero emissions in the coming decades. The International Energy Agency has estimated that a global net zero target will require CO_2_ emissions associated with buildings to decline by over 95%.^1^ Globally, one third of energy use in residential buildings is for the purpose of space heating and a further quarter is for domestic hot water (DHW).^2^ A common emission reduction strategy for heating and DHW in Europe and worldwide is electrification of these energy services alongside the decarbonisation of electricity.^1,3,4^ This strategy is proposed for the UK,^5^ with the most efficient technology option being heat pumps.^6^

Electrification of heat presents several electricity system challenges. For example, in Great Britain widespread adoption of heat pumps and other electric heaters is estimated to increase electricity demand by up to 65% and peak demand by up to 115%.^7^ It also creates large seasonal variations in electricity demand^8^ and exacerbates existing daily variations^9^ which are not necessarily correlated with supply of renewable electricity. Furthermore, transmission and distribution grids will require increased capacity.^10^

These electricity system challenges are significant, but it is anticipated that the demand side can mitigate some of them via demand response, defined by Albadi and El-Saadany^11^ as “all intentional electricity consumption pattern modifications by end-use customers that are intended to alter the timing, level of instantaneous demand, or total electricity consumption”. In certain cases, these changes can be implemented by third parties (e.g. the grid operator). In conjuction with time of use tariffs, heating can be operated to minimise costs, at times of low demand and/or high renewable generation.^12^ At times of higher cost electricity (including times of peak demand), households may be incentivised to turn the heating down or off. It is likely that most homes will not have dedicated thermal or electrochemical storage, therefore the thermal storage in the building itself will be relied upon to keep the home warm.^13^

Heat pump demand response using the building fabric as passive thermal storage has been modelled to assess its technical and economic potential in different countries and contexts (see literature review below). However, little empirical work has been carried out in occupied homes to explore the implementation of demand response and explore key model assumptions, such as that heat pumps can easily switch on and off as required.

Dynamic time of use tariffs are now commercially available internationally and in the UK^1^ and have begun to be used by early adopters of heat pumps and electric vehicles. This presents an opportunity to gain early learnings from real homes and test assumptions about how demand response is controlled. These findings can be used to inform heat pump demand response offerings as they are more widely adopted.

Since there are many possible types of heat pump demand response serving different electricity system functions,^10,14^ in this paper we focus on peak shaving; specifically, reducing heat pump electricity demand during the current UK peak period of 4 p.m.–7 p.m. This article presents technical learnings from three case study heat pump homes each having programmed the heat pump or heating system to reduce demand during this peak period, and each using different control strategies.

The paper is structured as follows. Relevant literature is discussed, then the research approach is given and the case study data and analysis methods are presented. Findings are presented, and reflected upon in the Discussion. The Conclusion summarises the implications for future demand response programs.

## Literature

### Demand response modelling studies and their assumptions

The most common approach to studying residential heat pump demand response is modelling.^15^ A key task in modelling studies is to estimate the electricity demand, CO_2_ emissions and cost from implementing demand response in a single dwelling or group of dwellings compared to a counterfactual case without demand response, under the constraint of maintaining occupant comfort. Normally, a mathematical representation of the physical dynamic system is implemented, which predicts the operation of the heat pump in order to calculate the electricity use, and the evolution of the internal temperature in order to evaluate occupant comfort.^16–18^

Modelling studies of heat pump peak load reduction share some common features and high-level outcomes. They often predict that demand response decreases peak-time power consumption but results in an efficiency penalty;^19^ this is partially explained by heat pumps not running at maximum efficiency due to excessive cycling or modulation.^10,14,20^ They are not usually validated using empirical data from buildings operated under demand response conditions.^21^ Finally, they assume that external signals such as time of use tariffs are communicated perfectly to the heating system which can respond in the required way, although they often do not specify how this communication occurs nor the mechanism by which the instruction to modulate heat output is given to the heat pump.

### Demand response in practice–controlling the operation of the heat pump

In practice, demand response with heat pumps is usually implemented with a time-of-use tariff or alternative notice system reflecting periods of high grid congestion. Demand can be reduced during those periods either manually or automatically, and either by the householder or an external party.^22^ In some cases, the demand reduction is preceded by a period of increased power to pre-heat, charging the thermal mass of the building and increasing the internal air temperature.^23^ Most field studies of heat pumps with demand response use automated third party control; these technologies are now commercially available and it is likely that automated third party demand response will be the prevalent means as heat pump demand response is adopted more widely.^24^

Each different implementation of demand response requires a *demand response control strategy*. This refers to the mechanism by which a message is sent to the heat pump to change its operation. Externally automated demand response can occur via *direct load control*, able to access the heat pump controller and therefore switch the heat pump off or down, and what we term *semi-direct load control*, in which another part of the system such as an air temperature thermostat can be manipulated but the heat pump itself cannot be accessed. A commercial example of the former is Florida Power and Light’s On Call^25^ offering which switches air conditioning and other appliances off completely or the EVU-Sperre scheme^26^ in Germany which allows for energy suppliers to remotely turn off heat pumps. Examples of the latter are Homely^27^ and Austin Energy’s^28^ use of internet-connected thermostats to adjust air temperature setpoints. These are all contrasted to *indirect load control*, which relies on participants to intervene, incentivised via a monetary or other signal, with no external control over the household’s energy use.

Two field trials of heat pumps with automated demand response in real homes provide examples of unintended outcomes of different control strategies. Sweetnam et al.^15^ studied 31 English homes in which heat pump use was automated in response to (simulated) price signals. The controller was able to turn the heat pump on and off and control the flow temperature setpoint. The system was required to meet certain temperature setpoints at certain times of day but was allowed to pre-warm the building beforehand with no temperature constraints. Through interviewing occupants, it was found that this led to temperatures being too warm and noise levels too high overnight. Energy use during high price periods was higher than expected; this was partly due to occupants updating settings throughout the day which conflicted with the algorithm’s predictions and caused the heat pump to run during high price periods.

A commercial trial also undertaken in England by NEDO^29^ again used automated third party direct load control, directly switching heat pumps off during evening peak periods. No dedicated pre-heating took place but there was a limit on allowed internal temperature drop before the heat pump restarted (maximum drop of 2°C, and a lower limit of 18°C). These parameters were the same in all trial homes. It was found that when heat pumps restarted after the demand response period, the period termed by Arteconi et al.^30^ as the “recovery period”, a spike in aggregated electricity consumption manifested due to coincident high power compressor operation to warm the homes up again.

In summary, many of the assumptions in models of heat pump demand response rely on unspecified perfect controls systems with no unintended consequences and no occupant intervention. Existing empirical work has begun to show the problems with these assumptions and more work is needed to examine how control strategies work in real situations.

## Research questions and approach

### Technical case studies of heat pump demand response

This paper presents data and analysis from an empirical study aiming to explore how heat pump demand response works in practice. The limited empirical evidence in the literature (see above) focusses on aggregation of electricity shifted across homes and total demand response available. This paper takes a different approach, analysing and understanding the consequences of different demand response control strategies in individual homes in detail, including how the strategies interact with and are interpreted by the heat pumps, the heating systems and the buildings in which they are implemented. Therefore, a technical case study approach was chosen. Case studies are particularly useful for exploratory research that aims to examine in detail a phenomenon in its context-specific setting.^31,32^ When used to focus on technical aspects of the phenomenon they allow a detailed understanding of performance.^33^

Three cases are reported in this paper, and the research can be classified as a comparative case study according to Sovacool et al.^34^ The main independent variable varying between three cases is demand response control strategy, although the cases also differ in other ways, which must be accounted for in interpreting the outcomes of the demand response. The focus of this paper is how the physical (electrical, temperature) outcomes are shaped by the technical aspects of implementation. A parallel paper^35^ presents our research exploring householders’ experience, treating demand response from a social practice perspective instead of as a technical system.

The research aims to answer the research question: how did the three different demand response control strategies work in practice? With associated sub-questions:

• RQ1: To what extent did the heat pumps reduce their power consumption during the peak period, and why?
• RQ2: How did each demand response control strategy interact with the heat pump’s control strategy?
• RQ3: What were the consequences of demand response for the heat pumps’ main energy services: internal temperature and hot water provision?

### Incorporation of social data in technical case studies

Two main data streams are used in this paper: technical data collected via technical methods (monitoring of the heat pump and dwelling internal conditions), and technical data collected via social methods (participants describing their heat pump systems and the technical implementation of demand response). The full set of methods and data collected are described in the Data section below.

The incorporation of technical data collected via social methods is unusual in case study research in this field. Each case study home was occupied by an energy expert who was attempting to implement heat pump demand response for the first time, and as a result of COVID-19 limiting researcher access to the houses, the technical details of the heat pump and demand response operation were provided by interviewing these experts instead of researcher inspection. This approach provided a significant benefit in allowing the researchers to gain a level of detail and understanding of the system over time which would not have been possible from normal, occasional, researcher site visits to properties. For example, a regular email exchange between the experts and the research team during data analysis helped to better understand the operation of participants’ heat pumps during demand response. However, it led the research team to rely on the experts’ accounts of their systems, unlike the normal approach of researchers directly inspecting systems themselves in times where site visits are less restricted.^36,37^ A limited degree of validation of the experts’ accounts was possible by observing the monitored data, for example the experts reported the timing of their hot water heating which could easily be verified, but full cross-checking between social and technical data as normally recommended^38,39^ was not possible.

## Data and analysis

### Data

Data from three case study homes was collected from October 2020 to May 2021. Ethical approval was gained from UCL Ethics Committee and a standard risk assessment and COVID risk assessment were carried out. The case studies were selected based on convenience sampling of households known to the researchers to have heat pumps which they either turned down/off according to a time of use tariff or were prepared to do so for the purposes of the study. This recruitment strategy was used for two reasons: the rarity in the UK of heat pump homes participating in demand response, and the timing which coincided with the COVID-19 pandemic making recruitment of previously unknown participants difficult.

The selection of case studies was not intended to be representative of the UK population; the homes were all inhabited by at least one energy and buildings expert and employed different demand management control strategies. This provided an opportunity to investigate the operation of the heat pump in each home in more detail than would have been possible without expert insight. It is worth noting that because of the participants’ expertise, their experience with the project (such as the indoor conditions during demand response) might be different to that of other people. However, the focus of this paper is on the technical aspects of the project and these differences were considered as being unlikely to affect the results. All three homes had had heat pumps installed in the previous 6 months, replacing gas boilers, thus it was their first winter of heat pump heating. In all homes the intended electrical reduction at peak time, hereon termed “demand response” for the purposes of this article, was programmed in by the occupants, and was not automatically linked to the time of use tariff nor communicated to an aggregator or other external party. However, the demand response control strategies employed in this study are appropriate for implementation by an external party aiming to manage the system or aggregate demand response; the strategies did not require occupant intervention once they had been programmed, and the programming could have been carried out remotely using an Application Programming Interface (API). The heat pumps, heating system configurations and dwellings represented a spread of typical situations, and it is these aspects which are the focus of this paper and thus can present valuable learnings.

Three different demand response control strategies were implemented within the sample:

• *Air temperature setpoint reduction* (house A): Thermostatic radiator valves (TRVs) were programmed to lower their setpoint to 15°C during the demand response period. This strategy is commonly used in modelling studies and can be automated via technology platforms such as Homely. The setback value of 15°C was selected by the occupant for the following reasons. The occupant wanted the heat pump to stay off, and therefore chose a setback temperature that they thought the house would not drop down to. In the event that their assumption was wrong and the house did drop down to 15°C, they did not want it to drop even further below this, preferring instead that the heat pump resumed operation.
• *Flow temperature setpoint reduction* (house C): The space heating flow temperature setpoint was lowered during the demand response period by programming it to operate in an ‘economy mode’. This strategy was used in order to keep the heat pump on (and so maintain thermal comfort) whilst reducing electricity consumption.
• *Compressor block* (house B): The heat pump compressor was programmed to switch off completely during the demand response period. This strategy was used partly in order to mimic commercially available direct load control strategies and partly because the occupant found that attempting to program in other strategies (such as air temperature setpoint reduction) did not in fact stop the heat pump from running during the demand response period, due to the complexity of the heat pump’s internal logic.

In all homes the heat pump was programmed to heat DHW at certain times outside of the demand response period. In houses A and B the occupants had programmed this explicitly; in house C the use of the economy mode from 4–7 p.m. excluded the possibility of producing DHW.

Two of the households also programmed into their heating systems some days in which the heat pump was run without demand response, in order to provide a control dataset which could be used by the researchers to compare electricity consumption under demand response and normal heat pump operating regimes. The third household did not do this, so the control dataset for this house was taken to be the 3 h preceding the 4–7 p.m. period, excluding days in which DHW was produced by the heat pump during these hours.

Relevant details of the case study homes are given in Table 1.

**Table 1.** Characteristics of case study homes.

	House A	House B	House C
House type	End terrace	Detached	End terrace
House age	1905	2011	1936
Floor area	231 m^2^	153 m^2^	136 m^2^
Thermal mass (construction)	High (30 cm solid walls)	Medium (cavity insulated walls)	High (23 cm solid walls)
Location	South West England	East England	South East England
Occupants	Female adult, male adult (energy expert), one child	Female adult, male adult (energy expert)	Female adult, male adult (energy expert), 1–2 teenagers
Other building fabric efficiency information	Uninsulated walls, insulated loft and ground floor. Some work done to improve airtightness	Well insulated	Uninsulated walls and floors, insulated loft. Some work done to improve airtightness
Heat pump type	Ground source, inverter control	Air source, inverter control	Ground source, fixed speed single stage
New radiators?	No – existing radiators large	Yes	Some radiators replaced with fan assisted radiators
Buffer tank	4-pipe buffer	2-pipe buffer	4-pipe buffer
	90 L	40 L	500 L (approx. 1/3 space heating, 2/3 DHW)
Circulation	Two circuits (heat pump to buffer, buffer to radiators)	One circuit	Two circuits (heat pump to buffer, buffer to radiators)
How heat pump was controlled	Internal temperature + heating curve	Internal temperature + adaptive heating curve (adjusted according to the difference between the internal temperature setpoint and the measured internal temperature)	Heating curve
Heating schedule	Continuous (except when producing DHW) using different air temperature setpoints throughout the day	Off from 4–7 p.m.	Continuous (except when producing DHW) using two modes: Normal space heating, and economy (lower flow temperature)
		Otherwise continuous (except when producing DHW)	
DHW schedule	Early hours of morning	Late evening	Evening and overnight
Control data	6 weeks in Jan/Feb without demand response	Demand response programmed not to take place on fridays or sundays	Three hours preceding the 4–7 p.m. period, on days with no DHW production during this time

The homes were monitored to obtain technical data about the heat pump and the associated internal conditions. Due to the COVID-19 pandemic restricting researcher access to homes the participants received the equipment through a combination of in-person drop-offs and post; they then installed the sensors themselves and downloaded data from them approximately once per month. Participants emailed this data to the research team. The technical data collected is summarised in Table 2.

**Table 2.** Summary of technical monitoring undertaken in each home.

Variable	Sensor/instrument	Logging resolution
Indoor air temperature (5 rooms)	HOBO U12-O12 loggers	10 min
Outdoor air temperature		
Radiant temperature (1 room)	Globe thermistor	10 min
Internal surface temperature of external wall	Surface thermistor	10 min
Heat pump electrical power	Current transducer, converting to electrical power assuming power factor of 0.95 and voltage of 240 v (case a and C) or measuring current, voltage and real power (case B)	5 min
Heat pump heat output and flow temperature	Heat pumps’ own monitoring systems	1 min

Two interviews were carried out with each household over video call using Microsoft Teams. The purpose was twofold: to understand participants’ experiences of demand response, and to provide more detail on the technical operation of the heat pumps from the energy experts in each household. Social data collected for the former purpose is further explained and used in our parallel paper,^35^ whilst the energy experts helped fulfil the latter purpose, e.g. by contributing to Table 1 and by helping interpret the physical data.

### Extra data analysis: Heat pump electricity consumption for house C

The electricity monitoring for house C failed due to the current clamp (see Table 2) not logging properly, and thus no heat pump electricity data was available for winter 2020/21. Electricity is an important variable in the study of demand response and so a method was devised to retrospectively estimate the electricity consumption of the heat pump on a 5 minutely basis in this house. The prediction method used the heat pump’s own heat meter data, divided by the estimated coefficient of performance (COP) of the heat pump, to estimate electricity consumption.

The COP of the heat pump for winter 2020/21 was estimated using an empirical model relating COP to outside temperature and heat pump mode, made using data from the winter afterwards (2021/22). The current clamp used in deriving electricity consumption was logging correctly by this next winter and the researchers were also able to access the heat pump’s heat data, mode and the outside temperature and thus to derive an empirical relationship between them. By the winter of 2021/22, the participants were no longer participating in demand response, since their electricity tariff had changed and no longer incentivised reducing consumption during the UK evening peak period. However, in this house the demand response during 2020/21 had been implemented by changing the heat pump’s mode from “space heating” to “economy”, and in 2021/22 the participants had programmed the heat pump to change between these modes overnight. Therefore, it was possible to derive heat pump COP in space heating and economy modes at a range of outdoor temperatures using 2021/22 data. This yielded errors of 2% on the relationship between space heating COP and outdoor temperature, and 7% on the relationship between economy mode COP and outdoor temperature. It also showed clear COP differences between modes (see Appendix 1). This enables a reasonable estimate of heat pump electricity consumption in 2020/21 both within and outside demand response periods. The results were also checked by aggregation and comparison to the smart meter data, with reasonable agreement between predicted and actual electricity consumption.

Appendix 1 Presents the original heat data from 2020/21 and the empirical relationships observed between COP and outdoor temperature for different heat pump modes, in 2021/22.

### Analysis metrics

A variety of metrics describing level of demand response achieved exist in the literature; the most relevant metrics depend on the application.^30^ For the analysis in this paper, several power and energy metrics were adapted from Arteconi et al.^30^ to render them suitable for use with real data instead of simulation results.

• Electrical power reduction: electricity use in the 4–7pm period on days with demand response compared to control days. Since this was found to vary linearly with outdoor temperature, a standard outdoor temperature of 5°C was used.
• Temperature drop: reduction in indoor air temperature over the demand response period. Since the rate of heat loss depends on the difference between indoor and outdoor temperatures, a standard internal-external temperature difference of 15°C is used. Note that other studies (e.g. Arteconi et al.^30^) do not present a temperature drop but a metric describing thermal comfort change, however here we make no prior assumptions about the band of temperature perceived by occupants as comfortable during demand response events. See Martin-Vilaseca et al.^35^ for an analysis of the link between temperature drop and thermal comfort in the case study homes.
• Daily electricity consumption change due to demand response: this is calculated by statistically comparing the linear relationships between daily heat pump electricity use and outdoor temperature for control days and days with demand response. Constants and coefficients were tested for difference using hypothesis testing. Differences are given at 5°C external temperature.

## Findings

### Presentation of results

The three research questions in this paper are addressed in the three following sections. These sections all draw upon two different presentations of the outcomes of the demand response which are given below: a summary table of key metrics (Table 3) and detailed plots of the heat pump’s behaviour in each house on the same day, using timeseries data.

**Table 3.** Demand response metrics applied to case study homes.

	House A	House B	House C
Demand response control strategy	Air temperature setpoint reduction	Compressor block	Flow temperature setpoint reduction
Heat pump electrical power reduction (averaged over 3 h period at 5°C outdoor temperature) (kW)	1.11	0.38	0.85
Heat pump electrical power reduction (as above, expressed as percentage) (%)	87	90	56
Temperature drop (over 3 h period, at internal-external temperature difference of 15°) (°C)	1.07	0.62	0.34
Daily electricity consumption change due to demand response	Demand response lowered daily electricity consumption by 1 kWh (significant at *p* = .05)	No statistically significant change	Unable to determine – no days in dataset without demand response

Table 3 gives key metrics described in the Analysis metrics section.

**Figure 1.**
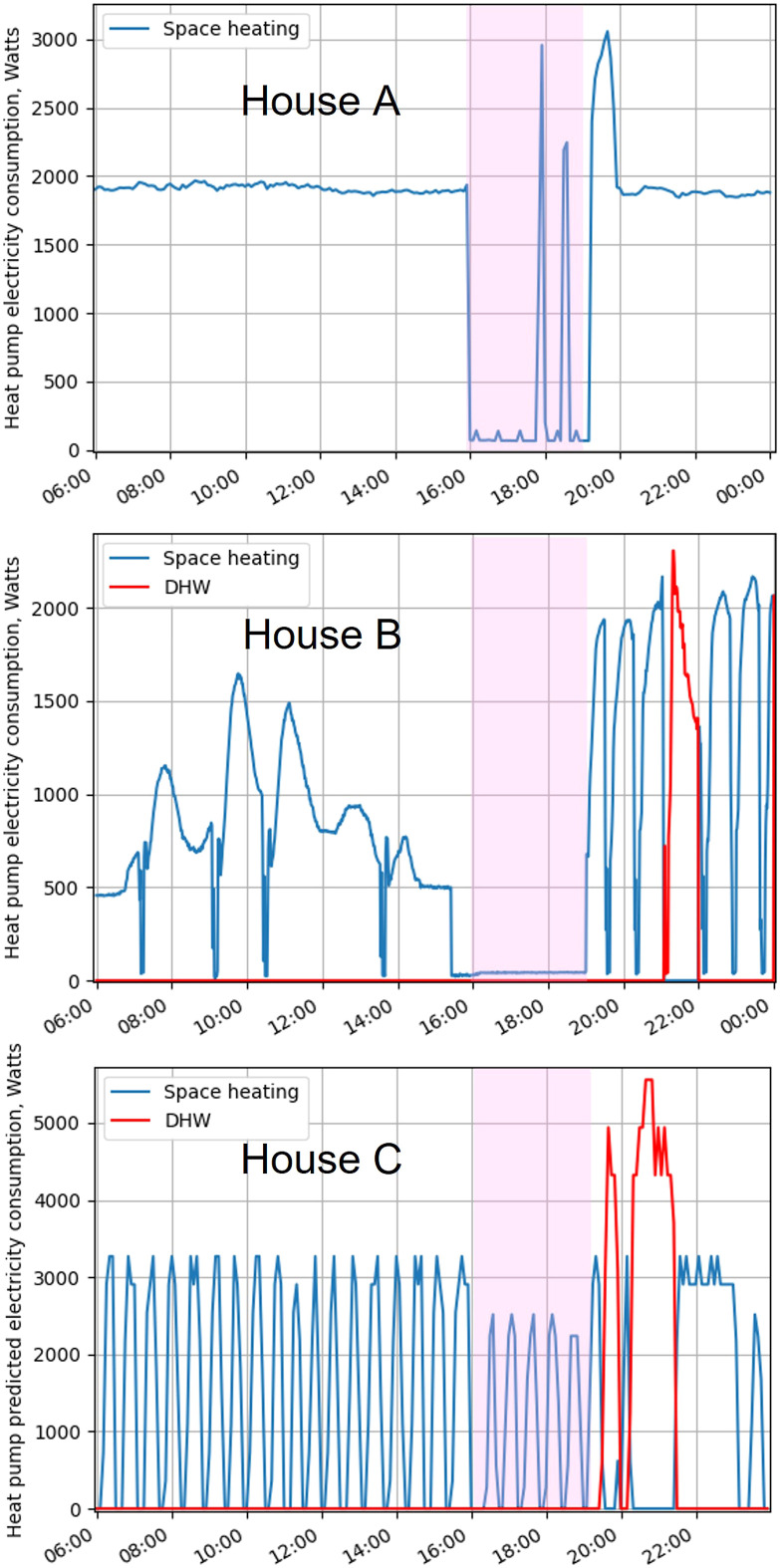
Heat pump electricity consumption (measured in houses A and B, predicted in house C), for 6 January 2021. Demand response period highlighted. Representing a typical cold winter day: Maximum outdoor temperatures of 2°C (a), 5°C (b) and 6°C (c).

Figure 1 shows the period before, during and after a demand response event in each house on a typical cold winter day (6 January 2021). The monitored timeseries electricity data is given for houses A and B, while in house C the predicted electricity is derived from the timeseries heat data in accordance with the procedure described above. Note that this prediction process correctly represents most features of the real power consumption of house C’s heat pump, such as the compressor cycle length and the approximate magnitude of the electricity consumption but distorts the variation in electrical power draw over one compressor cycle. Therefore, Figure 6 in the Appendix gives a plot from the following winter, showing monitored electrical power draw in different heat pump modes, to illustrate the real power profile over a cycle but outside of the context of demand response and the trial period.

### Reduction in heat pump power consumption during peak period

Table 3 shows that both houses A and B achieved large percentage reductions in electricity use during the 4–7 p.m. period. The compressor blocking strategy used in house B reduced the heat pump electricity demand to solely that of the parasitic electrical load comprising of heat pump standby, heat pump controls and other small loads.^40^ Due to the high energy efficiency of the house, this residual load constituted 10% of the heat pump’s electricity demand at an outdoor temperature of 5°C. While the electricity consumed by the circulation pumps is not included in the electricity demand, it is important to note that these pumps continued to run over the compressor block period at the 15% standby speed programmed in the controller and the occupant did not find a way to turn them off during the demand response period.

In contrast, the air temperature setpoint reduction strategy used in house A did not always prevent the heat pump compressor from running in the peak period; in such instances it initially turned off as expected but resumed operation before the end of the demand response period. This is illustrated in Figure 1 (top subplot), which shows short-duration electricity demand peaks of up to 3 kW during the highlighted demand response period. The reason for this behaviour is system and control design: the lack of direct linkage between the demand response signal and the heat pump. This house has two separately pumped hot water circuits involved in space heating: one linking the heat pump and buffer tank, and another linking the buffer tank and radiators (Table 1). The presence of an intermediate buffer tank meant that the chosen demand response message–in this case a reduction in air temperature setpoint at the radiators–does not communicate directly with the heat pump to stop producing heat but to the buffer tank and the circulation pump. The immediate effect of the change in set-point is for the circulation pump from the buffer tank to the radiators to switch off. The buffer tank sensor then detects that the tank temperature is no longer falling, signalling to the heat pump that heat was no longer required. However, even without the radiators calling for heat, heat loss from the tank over the three-hour demand response period was significant, causing the heat pump to operate to restore the buffer tank’s own setpoint.

The demand response strategy in house C was a space heating flow temperature reduction during the peak period, implemented by switching from ‘space heating’ to ‘economy’ mode. This temperature reduction was not fixed but varied with external temperature via the pre-programmed heating curves.^2^ This strategy resulted in a 43% reduction in measured heat output and a 56% reduction in predicted electricity consumption, with the heat pump still operating throughout the peak period. This continued operation was a consequence of controlling the heating using the heating curve, a steady state and feed-forward type of control. At the start of the demand response period, there was no signal from an air temperature thermostat indicating to the heat pump that it should turn off. Instead, the heat pump was simply instructed by its controller to operate at a lower flow temperature for several hours to keep the space heating flow at a reduced temperature. Thus, the heat pump continued to operate throughout the peak period, with reduced electricity consumption per compressor cycle as shown in Figure 1 (bottom sublot).

**Figure 2.**
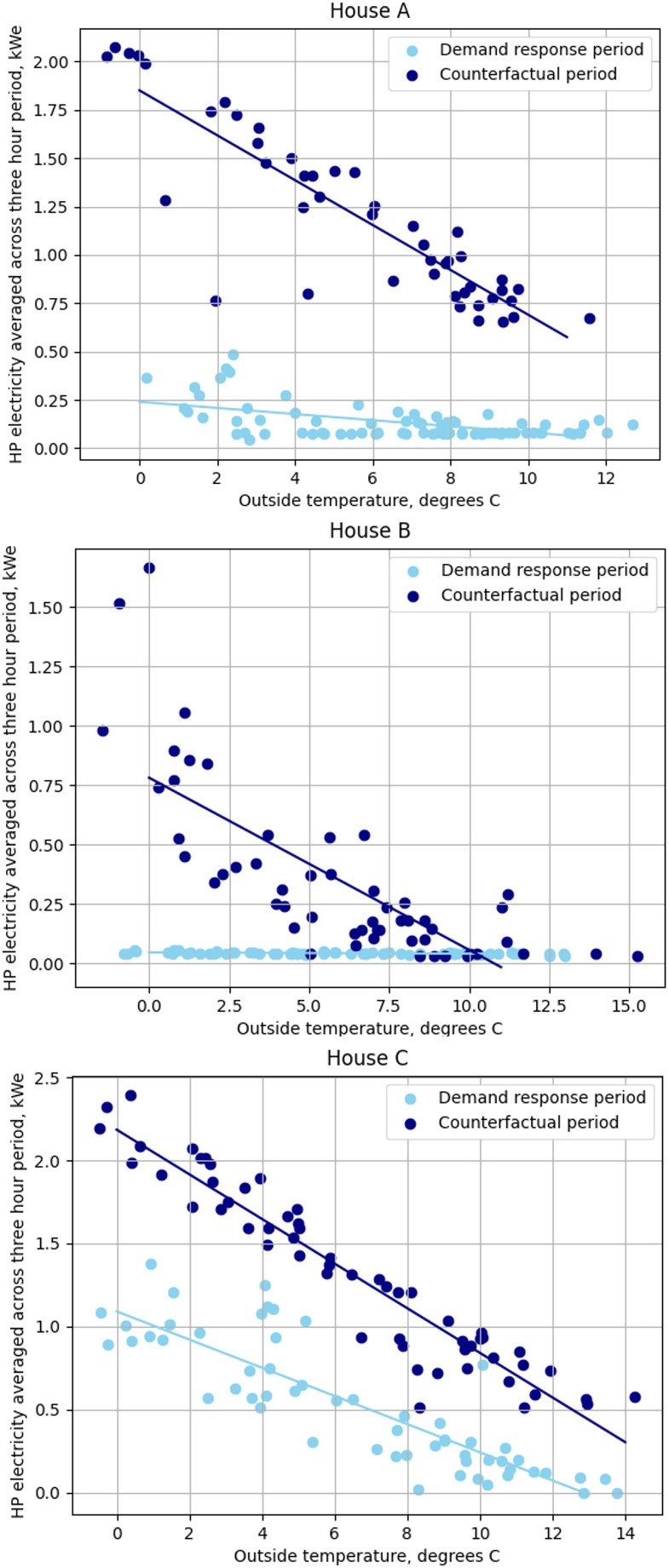
Heat pump electrical power reduction during the demand response period, over the entire monitoring period.

The reduction in electrical power across the whole monitoring period is shown for each house in Figure 2. It can be seen that more electricity is saved on colder days, but also that in the houses where the heat pump compressor operated during the peak period (A and C), electricity consumption during this time was higher on colder days.

### Interaction of demand response control strategy with heat pump control strategy

In two of the cases the demand response control strategy was intended to utilise inherent parts of the heat pump control strategy. Operation of the heat pump in house A was triggered by a drop in temperature of the water in the buffer tank, whose temperature was affected by the heat loss from the tank to supply radiators (and pipework) and from the tank itself. Thus, reducing the TRV setpoints during demand response was intended to indirectly signal to the heat pump to switch off (the previous section explained how this did not always occur in reality). The heat pump in house C was controlled by the outdoor temperature alone, via the heating curve: a pre-programmed relationship between the outdoor temperature and heat pump flow temperature designed to result in the specified indoor temperature. Lowering the space heating flow temperature during the demand response period was therefore intended only to reduce the heat pump output, not turn the heat pump off.

Conversely, in house B the demand response control strategy did not work within the heat pump control strategy. The heat pump was controlled by both the outdoor and the indoor temperature, also incorporating a “degree minute” control designed to prevent the heat pump cycling whilst keeping the temperature as close to the intended setpoint as possible. These algorithms were complex and “black box”, but their aim seemed to be to keep the heat pump running, as efficiently as possible, and adjusting the heating curve as needed to maintain the internal temperature. The energy expert had experimented with different ways of trying to stop the heat pump operating from 4–7 p.m. and had found that it kept coming back on. They had concluded that the only reliable way of implementing demand response was to use the heat pump controller to block the compressor operation.

#### Heat pump behaviour after the demand response period

The heat pump control strategies discussed above had important implications for the heat pump’s behaviour when the demand response period finished at 7p.m. Figure 1 illustrates the increase in heat pump power consumption occurring immediately after the 4–7 p.m. period in houses A and B. In house A this added around 50% to the typical power consumption for around 30–60 min. In house B it had a greater effect, with the power consumption doubling or more for several hours. During a very cold spell the electrical power consumption reached 3500 W, the heat pump’s maximum compressor rating, which may be detrimental to its longevity. The increased power consumption occurred because the controls called for high heat delivery to return the internal temperatures to setpoint at 7p.m. after a three-hour period of inactivity. However, in neither house did this increase the daily heat pump electricity consumption (Table 3).

It is worth noting that the typical diurnal pattern of external temperature means that it is most likely falling after about 16:00. The temperature recovery period after demand response therefore coincides with falling external temperatures and falling solar gains, thus even without demand response the system would potentially need to increase its output to achieve setpoint. At the same time, the performance of ground source heat pumps could benefit from the period of inactivity. The temperature of the water/brine in the ground loop will increase back up to the source temperature during the demand response period, reducing the temperature gap and therefore improving the COP of the system when the operation of the heat pump resumes. An initial analysis evidenced that this effect was small (COP increasing <0.5) and therefore this issue was not studied in more detail.

In house C the heat pump was required to heat DHW after the end of the demand response period so was not able to provide space heating to restore the desired internal temperature until 10 p.m. Furthermore, this was a fixed speed heat pump, unable to increase its instantaneous power output unlike those in houses A and B. However, the heat pump ran continuously from 10 p.m. until around midnight, without cycling off, indicating that its output was higher on average than prior to the demand response period.

### Consequences of demand response for the heat pump’s main energy services

**Figure 3.**
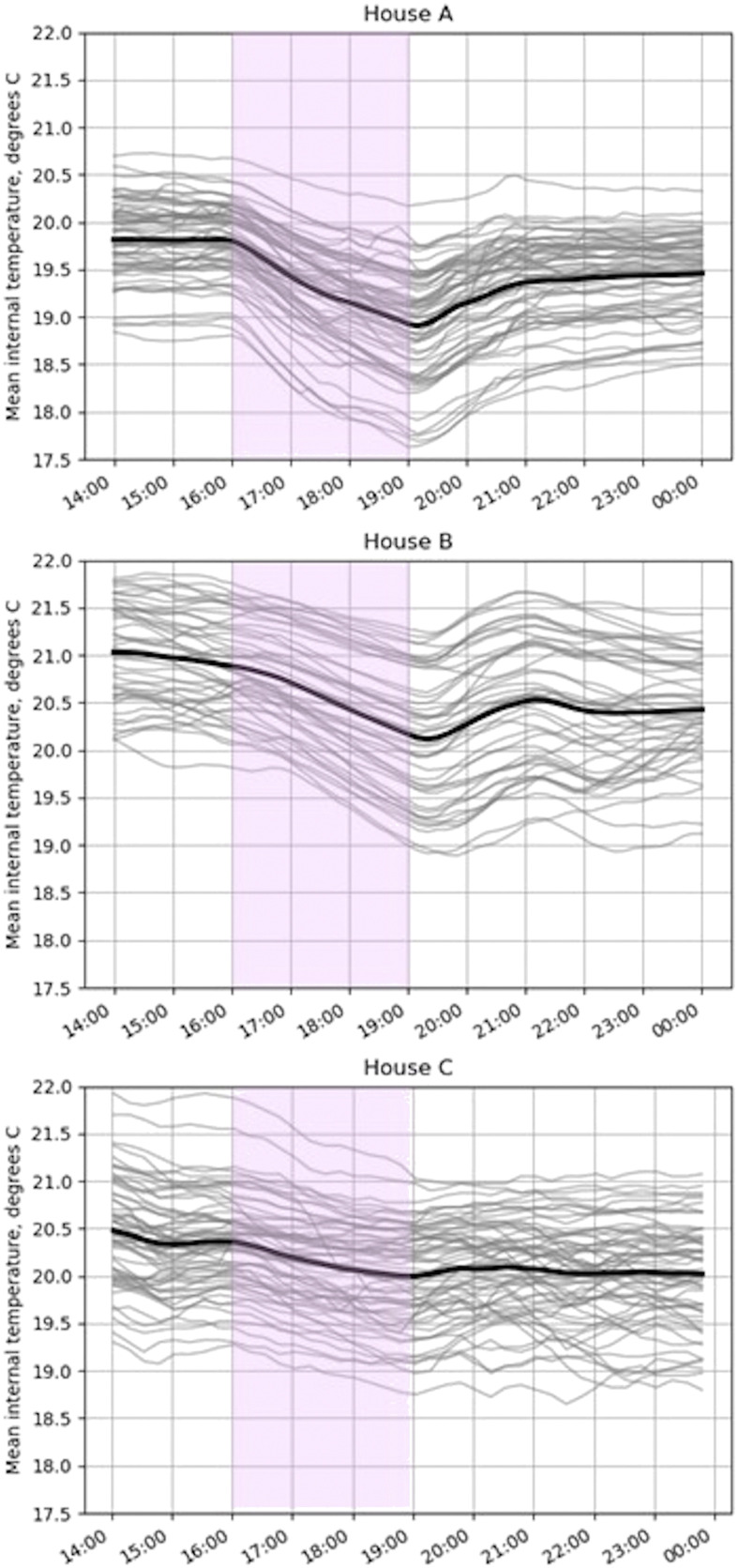
House temperature before, during and after the demand response period. Grey lines each show 1 day; note that different outdoor temperatures occurred each day and also between the homes. The single black line is the average over the monitoring period.

The mean internal temperatures of each home are shown in Figure 3. There are many factors which influence the temperature drop during the demand response period, including physical properties of the building, size of buffer tanks, outdoor temperature, internal gains and any heat pump operation. House C showed the lowest temperature drop; whilst this was an old house with uninsulated walls, the heat pump operated at around half its usual output during the peak period and this house had the largest space heating buffer tank. A small temperature drop also occurred in house B, a well-insulated house in which the heat pump did not run for the 3 hour period. House A, an old house with uninsulated walls, regularly cooled down more than a degree despite some heat pump operation during the peak period. In order to contextualise these temperature drops and comment on their acceptability, social data is required; this is explored in Martin-Vilaseca et al.^35^

The temperature evolution following the demand response period is assumed in modelling studies to rise back to the original setpoint,^30^ however in the case study homes this was not so straightforward. In houses B and C, the temperature recovery was interrupted by DHW demand (illustrated in red in Figure 1). In house A, the occupants set a lower internal temperature setpoint at 7 p.m., the end of the peak period, than that at 4 p.m., the start, due their belief that electricity prices were still high from 7 p.m. to 8 p.m. Thus, in none of the subplots in Figure 3 does the temperature return to its pre demand response level by midnight.

## Discussion

### Key findings and implications for implementation of heat pump demand response

The aim of this research was to explore how heat pump demand response works in practice, specifically focussing on the implementation and consequences of different demand response control strategies. It was found that the outcomes, in terms of reduction in heat pump operation during the demand response period and power increase afterwards, depend on how the demand response control strategy works with or against the control strategy and infrastructure of the rest of the heat pump/heating system. In this section, this issue is discussed and key learnings for future demand response implementation are highlighted.

#### Advantages and disadvantages of different demand response control strategies

This section refers to the terms introduced in the literature review: direct, semi-direct and indirect load control. For this research the demand response mechanisms were all programmed manually by the energy experts in each home and were all therefore indirect load control. However, we consider the implications for externally automated versions of the same strategies in response to time of use tariffs, as is widely expected to be adopted in the future. If externally automated, the compressor block and flow temperature setpoint strategies would come under the classification of direct load control strategies: an external party adjusting the heat pump controller, and the air temperature setpoint strategy would be classified as semi-direct load control: the external party able to access an air thermostat but not the heat pump.

Different control strategies were found to lead to different amounts of demand response, from complete load shedding to continued heat pump operation at lower heat pump output. The success of each strategy depends on what the aim is–total or partial load shedding–and also for how much of the peak period this needs to occur.

The only control strategy which reliably resulted in the heat pump staying off throughout the entire peak period was the direct load control strategy of blocking the compressor. Semi-direct load control via air temperature setpoint did not ensure that the heat pump stayed off, especially as part of a two-circuit heat pump configuration with buffer tank, where the thermostat does not control the heat pump directly. Instead, it had the effect of delaying heat pump operation until some way into the peak period when the buffer tank cooled down and called for heat from the heat pump, to maintain its setpoint. This control strategy could lead to significant reduction in total energy use in the demand response period but does not guarantee that loads will not present to the system. Finally, reducing the flow temperature setpoint reduced energy consumption by around half in this case, but did not eliminate heat pump operation; it is a partial demand reduction strategy, but could lead to high bills for customers due to operation during peak tariff times, unless accounted for in tariff structures. In theory this strategy could be used to switch off the heat pump completely during the demand response period, but the project did not explore if this would work in practice.

#### Interaction between demand response control strategy and heat pump technology/rest of heating system

There are different options for how to reduce heat pump load during a certain period, of which this study considered three. A key lesson from this work is that the intended effects may not be achieved unless thought is given to how the demand response control strategy works with the control logic of the heat pump and overall design of the heating and hot water system. Two examples are used here to illustrate this issue.

In house A, the intention was for the room air temperature thermostat setting to ensure that the heat pump stayed off for the full demand response period. This did not occur, because the heat pump was controlled by the buffer tank setpoints, not the room thermostat directly, and the buffer tank was able to call for heat from the heat pump without a signal from the room thermostat.

In all homes the average power consumption increased after the demand response period, but this was most problematic in house B, which had used a compressor block demand response strategy. This heat pump responded by working very hard to restore the internal temperature after a 3 hour period of the house cooling down, resulting in high electricity consumption over the next hour as observed in the NEDO trial,^29^ which could have detrimental effects to its compressor and could be problematic if it occurred on a large scale. If direct load control strategies are used at scale, heat pumps’ internal algorithms may benefit from redesign to deliver a curtailed heating power when raising temperature after the demand response period. Such a mechanism could be combined with management of the tariff signals for peak demand periods across the stock, to avoid a large post-demand-response peak in demand. The former curtailment mechanism would also be beneficial if operation at or close to maximum compressor output is found to be detrimental to heat pump service life, and thus mitigating potential compressor wear or damage.

The above two examples show the need to coordinate the demand response control strategy with the heat pump logic and the rest of the heating system. Although this may sound obvious, it is not clear how this will be carried out in practice when the party implementing the demand response (e.g., an aggregator or an energy company) may not know or be able to access all of the necessary controls or information, whilst the internal algorithms of the heat pump are often proprietary and opaque. Heat pumps with different control logic are installed in many different configurations with respect to the rest of the heating system, and demand response strategies will have to work with this heterogeneity amidst limited knowledge and control. As a minimum, this will require standardising communication using a protocol such as CHAdeMO in electric vehicle charging, the EVU-Sperre in Germany^41,42^ or the OpenADR,^38^ implemented in California and currently being tested in other countries.

### Limitations of study and future work

This technical field trial of three case studies did not aim to discover all possible outcomes from heat pump demand response but to illustrate some cross-cutting issues using examples. It did not cover all types of dwelling from a physical perspective–there were no inefficient, thermally lightweight homes; nor all demand response control strategies–for example there were no instances of ‘smart’ solutions remotely optimising heat pump operation to preheat before the demand response period.

There is much more technical analysis which could be undertaken using the case study data, for example heat pump COP analysis and the role of the heating system thermal mass including the buffer tank in maintaining the air temperature. However this paper limits its scope to comparing the three different demand response control strategies and examining their effect on electricity reduction and provision of energy services during the peak period, since little work on this can be found in previous literature.

This paper did not explore how occupants perceive and interact with the system, for several reasons. Firstly this requires a very different ontology, able to combine the monitored variables with social perspectives which are not researched through the same units, deeming a separate article more suitable.^35^ Secondly our study was limited in its socio-demographic representation, since each home was occupied by at least one expert in energy and/or heat pumps. This benefitted this paper since these experts were able to provide technical information on the operation on the system to give a rich knowledge of how the components operated and interacted, to enhance and understand the technical data. This type of data is very useful for case studies of complex multi-technology systems in the field. The technical aspects reported in this paper are not affected by the expertise of the participants. However, the thermal perception of these participants and their experience of DSR are likely to be different from the experience of other people. For that reason, the accompanying socio-technical paper is also reporting on the interviews with the other adults living in the household.^35^

The next step for future research is a large scale socio-technical trial of demand response, potentially incorporating technical interventions to poorly performing systems,^39^ and specifically investigating how demand response fits into household everyday life. This will help quantify the realistic potential of demand response as opposed to the technical potential output from modelling studies. It will also give useful insight into which demand response control strategies are most suitable for different household and building types.

## Conclusion

Electric heat pump installation is expected to accelerate in several countries to decarbonise heating, whilst the move to low carbon electricity is reducing the flexibility of generation. Increased energy flexibility from the demand side, through the demand management of heat pump operation, has become a potentially important component of ensuring energy system resilience at low cost.^10^ Whilst much modelling has been undertaken, few studies have explored the real performance of heat pumps within homes,^15^ and the consequent opportunities and constraints this places on the availability of demand response.

In this paper, data and analysis has been presented from three case studies of early adopters of heat pumps with demand response, each implementing this using a different system set up but with a common aim of reducing electricity consumption over the same three-hour period. The three strategies are all viable commercial automation strategies.

Which strategies are most “successful” depends on the aims for the electricity system and the household. If total load shedding is required, it is likely that room temperature setpoint changes will not guarantee this, even if these setpoints are very low; it was seen here that calls for heat from the heat pump are caused by other system components. Shutting down the compressor is of course guaranteed to keep it off for the required duration (except for certain parasitic loads); this can potentially lead to very high heat pump load immediately after the demand response period, depending on the internal logic of the heat pump. Heat pumps are most efficient when run at low flow temperatures and in constant operation; if total load shedding is a likely future requirement, heat pump controller design will have to anticipate this and restart after the peak period in a way which does not place strain on the compressor or, at a large scale, the electricity grid. If partial load shedding is sufficient, heat pump flow temperature reduction may be an effective strategy which fulfils this aim yet slows the temperature drop of the dwelling and eases the need for heat pumps to compensate after the end of the critical peak period.

The technical success of heat pump demand response was also shown to require integration of the control strategy with the rest of the heating system components within the dwelling, as well as on the grid side which is more commonly discussed. This integration affected the amount of demand response provided and the electricity consumed during the recovery period, among other issues. However, this issue has been poorly acknowledged in the literature and there is not a clear vision of how the integration should be delivered. The heterogeneity of domestic heating systems makes it difficult to find a one-fits-all solution and the complexity of those systems, usually made up of several parts involving different stakeholders (e.g., hydronic system, heat pump, buffer tank, etc.), means that it is not clear who should be responsible for delivering an integrated system to support demand response. The experiences outside the UK or for other technologies could provide useful approaches to this challenge. For example, in Germany, this issue has been addressed at several levels: from the heat pump manufacturers (who are designing heat pumps to be prepared to receive and interpret ripple signals) to the energy providers (who offer low-price electricity tariffs for households with heat pumps that allow certain external control of the system).^26^

The effect of heat pump demand response on the heat pump’s main energy services highlighted that DHW provision is an important consideration. The occupants in this study valued having hot water in the evening more than a fast internal temperature recovery; this finding is not necessarily generalisable but indicates that some occupants do use the heat pump to generate DHW in the evening where it is normally assumed that this is carried out at other times of day or night. When DHW heating is occurring the heat pump cannot deliver space heating and therefore cannot recover from any temperature drops caused by demand response.

This study has highlighted the importance of empirical work in uncovering cross-cutting issues associated with practical heat pump demand response. Understanding the real-world potential of demand response from a quantitative perspective requires further work at a larger scale, in households without experts, in a range of building types, with different heat pumps installed in different heating system configurations. This must be co-developed with the requirements of the future electricity system, to create effective strategies to deliver sufficient levels of demand response from a large future load on the electricity grid.

## Supplemental Material

Supplemental Material - Demand response with heat pumps: Practical implementation of three different control options Click here for additional data file. Supplemental Material for Demand rresponse wwith hheat ppumps: Practical iimplementation of tthree ddifferent ccontrol ooptions by Jenny Crawley, Adria Martin-Vilaseca, Jez Wingfield, Zachary Gill, Michelle Shipworth and Clifford Elwell in Building Services Engineering Research and Technology

## Notes

a. See for example https://octopus.energy/agile/

b. For example, at an outside temperature of 3°C on 1 January 2021 the space heating flow temperature reduced from 36°C to 28°C, and at an outside temperature of 8°C on 5 February 2021 it reduced from 44°C to 37°C.
