# Supplementary material for: Demand response with heat pumps: Practical implementation of three different control options
Source: Build Serv Eng Res Technol. 2022 Dec 13;44(2):211–28. doi: 10.1177/01436244221145871 (PMC9976642; doi:10.1177/01436244221145871)
Supplement: Supplemental Material - Demand response with heat pumps: Practical implementation of three different control options [file sj-pdf-1-bse-10.1177_01436244221145871.pdf]

Appendix 1

Figure 1, Figure 2 and Figure 3 supplement the extra analysis described in Section 4.2 to estimate heat pump electricity consumption in house C where the current clamp did not log properly during the trial period.

[insert Figure 4.]

Figure 1. Heat pump heat output, house C.

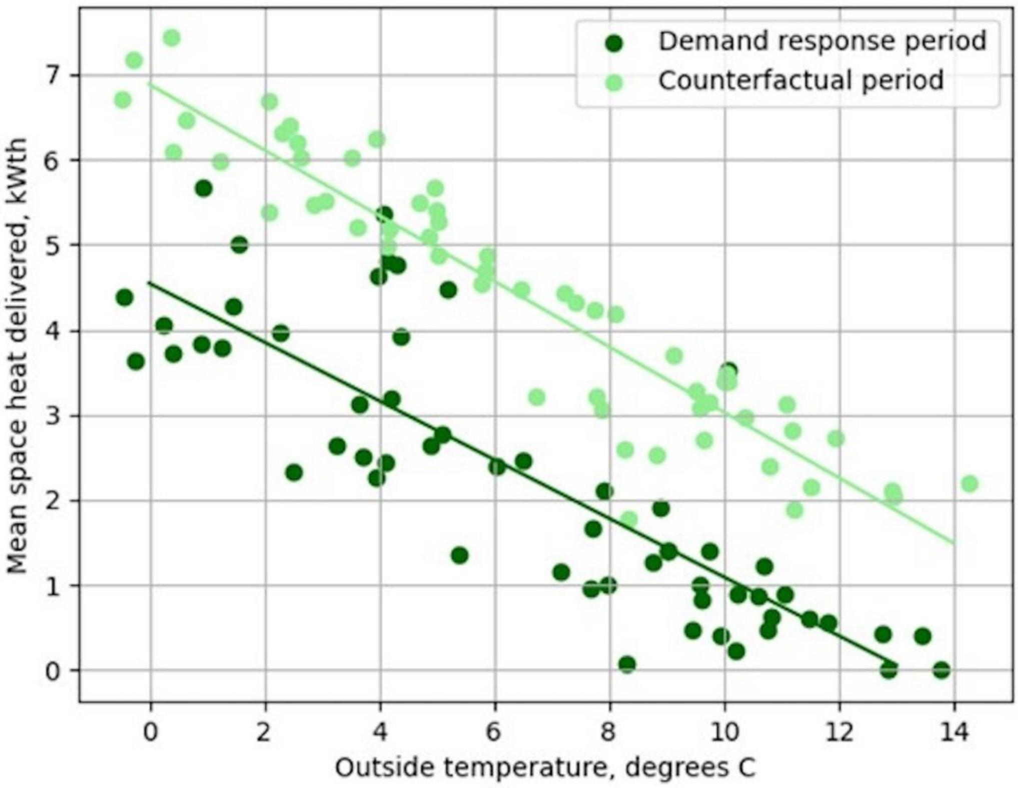

[insert Figure 5.]

Figure 2. Empirical relationships between COP and outdoor temperature for different heat pump modes, house C, winter 2021/22.

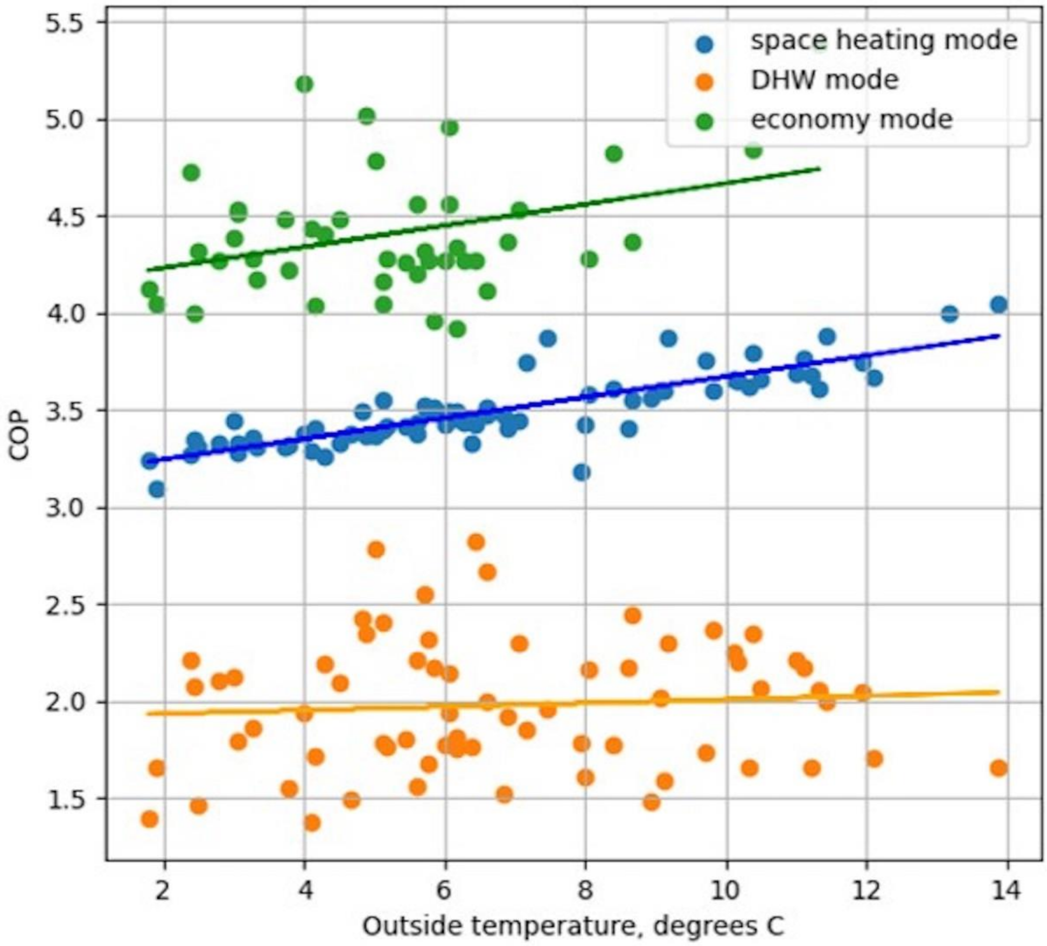

[insert Figure 6.]

Figure 3. Monitored electricity consumption, house C heat pump, example winter day (16/17 January 2022).

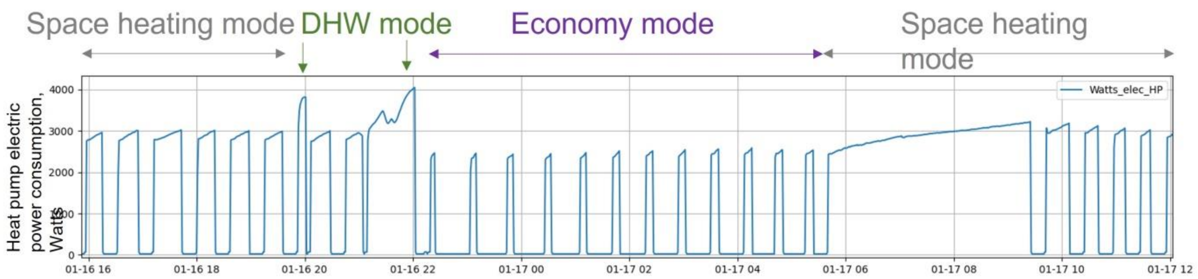

Figures

Figure 4. Heat pump heat output, house C...1

Figure 5. Empirical relationships between COP and outdoor temperature for different heat pump modes, house C, winter 2021/22...1

Figure 6. Monitored electricity consumption, house C heat pump, example winter day (16/17 January 2022)...2
